# Network Analysis of Emotional Labor, Emotional Display Rules, Perceived Organizational Support, and Emotional Intelligence Among Nurses

**DOI:** 10.1155/jonm/4202514

**Published:** 2026-08-19

**Authors:** Xu-Dong He, Xiao-He Lin, Xiang-Yu Zhao, Xiao-Na Shen, Guo-Peng Li, Ping Li

**Affiliations:** ^1^ School of Nursing and Rehabilitation, Shandong University, 44 Wenhua West Road, Jinan 250012, Shandong, China, sdu.edu.cn

**Keywords:** emotional display rules, emotional intelligence, emotional labor, network analysis, nurse, perceived organizational support

## Abstract

**Background:**

Nurses are required to manage their emotional expressions to meet professional standards and provide optimal support. Emotional labor has become an inevitable part of their daily professional lives. Understanding the relationships between emotional labor and its antecedents is crucial for guiding nurses to adopt beneficial emotional labor strategies, thereby enhancing their well‐being and the quality of care.

**Aim:**

To examine the interdependence among emotional labor strategies, emotional display rules, emotional intelligence, and perceived organizational support among clinical nurses using network analysis, and to identify central and bridge components that may inform future nursing management research.

**Methods:**

A secondary analysis was conducted using cross‐sectional data from 2996 nurses across eight cities in Shandong Province, China. Data on emotional labor, emotional display rules, emotional intelligence, and perceived organizational support were collected. We estimated an undirected network using a Gaussian graphical model (GGM) and employed Bayesian network analysis to infer potential directional associations, visualized as a directed acyclic graph (DAG).

**Results:**

The undirected network identified “Use of emotion” and “Deep acting” as the nodes with the highest expected influence, indicating their strong connectivity with other components of the emotional labor network. “Positive display rules” exhibited the highest bridge expected influence, functioning as a link between the organizational norms and individual emotion regulation. Additionally, in the estimated DAG, “Self‐emotion appraisal” occupied a relatively upstream structural position, providing an exploratory indication of potential directional organization.

**Conclusions:**

This study provides preliminary insights into the network structure linking emotional labor, emotional display rules, emotional intelligence, and perceived organizational support among clinical nurses in Shandong Province, China. “Use of emotion,” “Self‐emotion appraisal,” and “Positive display rules” emerged as structurally important components that warrant further examination in longitudinal and intervention studies. The findings may be most applicable to clinical nurses working in similar Chinese tertiary hospital contexts.

**Implications for Nursing Management:**

In similar Chinese tertiary hospital contexts, nursing managers may consider fostering supportive work environments and developing flexible, noncoercive positive display norms that support adaptive emotional regulation and the appropriate expression of naturally felt emotions. Emotional utilization and self‐emotion appraisal may also represent relevant areas for future management programs; however, these implications are based on exploratory cross‐sectional evidence and require confirmation in longitudinal or intervention studies.

## 1. Introduction

Driven by the reforms of healthcare institutions and the growing societal awareness of patient rights, the demands placed on healthcare professionals have increased [1]. As a critical component of the medical workforce, nurses are tasked not only with navigating complex clinical challenges but also with providing emotional support to patients and their families. To meet these public expectations, nurses must coordinate efficiently with medical teams while delivering compassionate care [2]. These demands necessitate that nurses effectively regulate their emotional expressions to align with role expectations, making emotional labor an inevitable part of daily nursing practice [3]. However, this invisible labor tends to be overlooked in clinical settings [4].

Emotional labor involves the management of feelings and expressions to fulfill organizational expectations [5]. It is typically manifested through three distinct strategies: Surface Acting, Deep Acting, and the Expression of Naturally Felt Emotions [6]. Surface Acting involves displaying false emotions or suppressing genuine ones, whereas deep acting requires actively modifying one’s internal feelings to align with the required expressions. In contrast, the expression of naturally felt emotions represents a state of spontaneous congruence, wherein nurses genuinely experience and express professional emotions without deliberate regulation [6]. Empirical literature suggests that distinct strategies yield different outcomes: Surface Acting is associated with negative outcomes such as burnout and turnover intention [4, 7], while deep acting is often linked to positive emotional experiences, enhanced job satisfaction, and superior care quality [2]. Moreover, the expression of naturally felt emotions has been shown to enhance both personal well‐being and organizational performance [3, 8]. Therefore, understanding the antecedents of each emotional labor strategy is crucial for guiding nurses toward more adaptive emotional regulation.

Based on Grandey’s emotional regulation model, emotional labor is influenced by three primary factors: interaction expectations, individual factors, and organizational factors [9]. Existing literature identifies emotional display rules, emotional intelligence, and perceived organizational support as the pivotal antecedents corresponding to these three factors [4, 10, 11]. Specifically, emotional display rules represent interaction expectations, serving as organizational norms that regulate appropriate emotional expression within the workplace [12]. In the nursing context, the profession’s inherent nature—characterized by the delivery of compassionate and patient‐centered care—subjects nurses to particularly rigorous display rules [13]. To align with these expectations, nurses must continuously engage in emotional labor. Hwang et al. reported that higher levels of display rules were positively associated with increased engagement in emotional labor among nurses [13]. As a vital internal resource, emotional intelligence refers to the capacity to monitor and regulate emotions, traditionally comprising four dimensions: Self‐Emotion Appraisal (SEA), Others’ Emotion Appraisal (OEA), Use of Emotion (UOE), and Regulation of Emotion (ROE) [14, 15]. Individuals with high emotional intelligence are not only capable of effectively managing their own affective states but also adept at identifying and evaluating emotional cues within social interactions, thereby facilitating the adoption of adaptive regulation strategies [16, 17]. Furthermore, perceived organizational support represents nurses’ beliefs regarding the extent to which the organization values their contributions and cares about their well‐being. As a critical external resource, perceived organizational support provides both instrumental and emotional backing, thereby increasing the resources and effort nurses are willing to invest in emotional labor. For instance, Chou et al. found that nurses perceiving higher organizational support were more inclined to modulate their internal feelings to meet organizational demands, predominantly employing deep acting rather than surface acting [18].

Although previous research has explored the relationships between these antecedents and emotional labor, most studies have treated these factors in isolation, largely overlooking their interdependence and synergistic impact. Evidence suggests that these antecedents are closely interlinked. For instance, Cai et al. suggested that higher organizational support may enrich individuals’ psychological resources, thereby enhancing their capacity to identify, regulate, and express emotions [19]. Similarly, Wen et al. found that perceived organizational support moderated the mediating role of deep acting in the relationship between emotional intelligence and job satisfaction [20]. However, traditional statistical approaches often fail to identify the components most closely associated with emotional labor when accounting for the interplay of these antecedents. Additionally, since distinct emotional labor strategies yield different outcomes, it is crucial to examine them as separate dimensions. Surface and deep acting are often regarded as compensatory strategies employed when individuals cannot spontaneously generate required emotions [6]. In contrast, the expression of naturally felt emotions allows individuals to meet organizational display rules without deliberate regulation, representing a resource‐conserving strategy [21]. Despite its importance, existing literature has predominantly focused on surface and deep acting, with relatively little attention being paid to the expression of naturally felt emotions [22].

Network analysis provides a useful approach to address these gaps by modeling emotional labor and its related factors as an interconnected system rather than as isolated predictors and outcomes [23, 24]. Unlike variable‐centered approaches, such as regression, mediation, or structural equation modeling, which usually estimate independent associations or prespecified pathways, network analysis allows us to examine which components are most strongly connected within the emotional labor system and which nodes bridge organizational, interpersonal, and individual domains. This approach is particularly relevant for the present study because emotional display rules, emotional intelligence, perceived organizational support, and different emotional labor strategies may mutually interact in clinical nursing contexts. By identifying central nodes, bridge nodes, and exploratory directional tendencies, network analysis can help clarify the interdependence among these constructs and generate hypotheses for future longitudinal and intervention studies.

**FIGURE 1:**
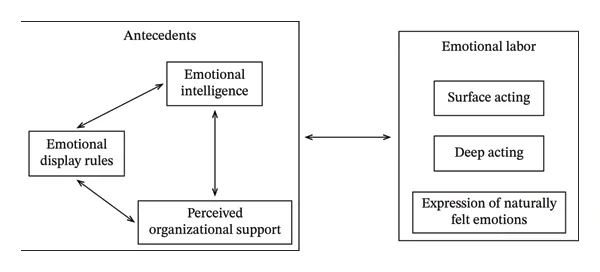
The hypothesized conceptual framework of this study.

Therefore, this study adopted a network perspective to examine the complex interrelationships among emotional labor strategies, emotional display rules, emotional intelligence, and perceived organizational support among clinical nurses. Specifically, we first estimated an undirected network to identify the most strongly connected components and bridge nodes across theoretical domains. We then constructed a directed acyclic graph (DAG) to explore probabilistic orientation tendencies among these components. The hypothesized conceptual framework is shown in Figure 1. The findings are expected to provide preliminary network‐informed evidence for understanding nurses’ emotional labor and for guiding future research and nursing management practices in similar clinical contexts.

## 2. Methods

### 2.1. Design and Participants

This study was a secondary analysis of cross‐sectional data collected from clinical nurses in Shandong Province, China, between June and December 2021 [25]. The original study used a multistage sampling procedure. First, the 16 prefecture‐level cities in Shandong Province were stratified into four geographical and socioeconomic regions: eastern Shandong, central Shandong, western Shandong, and south‐central Shandong. Two cities were randomly selected from each region, resulting in a total of eight participating cities. Hospitals within the selected cities were purposively recruited through existing research collaboration networks. The nursing administration departments of participating hospitals distributed the electronic questionnaire to clinical nurses who met the eligibility criteria. Eligible nurses were invited to participate voluntarily and indicated their informed consent by completing and submitting the online questionnaire. The inclusion criteria were (1) being a registered nurse currently engaged in clinical practice; (2) having more than 6 months of work experience; and (3) providing voluntary consent to participate. The exclusion criteria were (1) intern nurses and (2) nurses who were on leave for any reason during the survey period. A total of 3037 questionnaires were submitted. Of these, 41 questionnaires were excluded because they were completed in less than five minutes. Consequently, 2996 valid questionnaires were retained for the final analysis, yielding a valid questionnaire rate of 98.7%.

### 2.2. Measures

The data were collected using a self‐administered, structured questionnaire including a study‐specific section on sociodemographic and occupational variables, as well as scales measuring emotional labor, emotional display rules, emotional intelligence, and perceived organizational support.

#### 2.2.1. Demographic and Occupational Variables

Participants’ age, gender, marital status, education level, income status, work experience, hospital level, work shift, and department were collected using a self‐developed questionnaire.

#### 2.2.2. Emotional Labor

Emotional labor was measured using the Emotional Labor Scale (ELS) developed by Diefendorff et al. [6]. The scale comprises three dimensions: Surface Acting (7 items, e.g., I put on an act in order to deal with patients in an appropriate way), Deep Acting (4 items, e.g., I try to actually experience the emotions that I must show to patients), and the Expression of Naturally Felt Emotions (3 items, e.g., The emotions I express to patients are genuine). Items are rated on a 5‐point Likert scale, ranging from 1 (*strongly disagree*) to 5 (*strongly agree*). A score for each strategy was calculated by summing the relevant items, with higher scores indicating greater engagement in that strategy. Its validity has been well established in older Chinese populations [26]. In this study, the Cronbach’s *α* coefficients for Surface Acting, Deep Acting, and the Expression of Naturally Felt Emotions scales were 0.83, 0.88, and 0.86, respectively.

#### 2.2.3. Emotional Display Rules

Display rule perceptions were assessed using the 7‐item scale developed by Diefendorff et al. [6], which comprises two dimensions: positive display rules (4 items) and negative display rules (3 items). Items are rated on a 5‐point Likert scale, ranging from 1 (*strongly disagree*) to 5 (*strongly agree*). Dimension scores were calculated by summing the respective items, with higher scores indicating stronger perception of the corresponding rules. The Chinese‐version scale has satisfactory psychometric properties [27]. In this study, the Cronbach’s *α* coefficients were 0.928 for positive display rules and 0.926 for negative display rules.

#### 2.2.4. Emotional Intelligence

Emotional intelligence was measured using the 16‐item Wong and Law Emotional Intelligence Scale (WLEIS) [28]. It assesses four components: SEA, OEA, UOE, and ROE. Items are rated on a 7‐point Likert scale ranging from 0 (*completely disagree*) to 6 (*completely agree*). Dimension scores were calculated by summing the relevant items, with higher scores indicating greater competence in that domain. Its validity has been well established in older Chinese populations [29]. In this study, the Cronbach’s *α* coefficients for SEA, OEA, UOE, and ROE were 0.889, 0.894, 0.895, and 0.930, respectively.

#### 2.2.5. Perceived Organizational Support

Perceived organizational support was measured using the scale developed by Zuo et al. [30], which consists of two dimensions: emotional support (10 items) and instrumental support (3 items). Items are rated on a 5‐point Likert scale from 1 (*strongly disagree*) to 5 (*strongly agree*). Scores for the two dimensions were calculated separately, with higher scores indicating stronger perceived support. In this study, the Cronbach’s *α* coefficients were 0.972 for emotional support and 0.932 for instrumental support.

### 2.3. Ethical Considerations

The original study was approved by the institutional review board of the School of Nursing and Rehabilitation, Shandong University, Jinan, Shandong Province, China (2020‐R‐135), and the approval covered subsequent analyses of anonymized data. Participants were informed that their de‐identified data could be used in future related research, and completion and submission of the online questionnaire indicated informed consent. The present secondary analysis involved only anonymized data and no additional contact with participants.

### 2.4. Statistical Analysis

#### 2.4.1. Descriptive Analysis

Descriptive analysis was conducted in SPSS Version 27.0. Continuous variables were presented as mean (*M*) and standard deviation (SD), while categorical variables were presented as frequency (*n*) and percentage (%).

#### 2.4.2. Network Analysis

Network analysis was performed using R software (Version 4.3.3). We constructed both an undirected network and a DAG to explore the complex interrelationships among emotional labor, emotional display rules, emotional intelligence, and perceived organizational support. The network comprised 11 nodes, each representing the summed score of a subdimension and being modeled as a continuous variable. Using summed scores as nodes is a common practice in network analysis, offering practical interpretability and stable estimates, particularly with large sample sizes [31]. Based on Grandey’s emotional regulation model, these 11 nodes were grouped into four theory‐driven communities: emotional labor (Surface Acting, Deep Acting, and Expression of Naturally Felt Emotions), emotional display rule perceptions (positive and negative display rules), perceived organizational support (emotional and instrumental support), and emotional intelligence (SEA, OEA, UOE, and ROE). Prior to network estimation, distributional properties were examined. The absolute values of skewness for all variables were below 1, and kurtosis values were below 4, indicating an approximately normal distribution [32].

Because hospital‐ and city‐specific identifiers were not retained in the anonymized dataset, clustering at the individual hospital or city level could not be directly assessed. As an exploratory assessment of between‐group variation, intraclass correlation coefficients were calculated across hospital‐level categories and department categories for all 11 study variables. All ICC values were below 0.01, indicating minimal variation across these observed categories [33]. However, these analyses do not rule out residual clustering within specific hospitals, cities, or clinical units. The network analysis was conducted at the individual level, and the potential influence of unmeasured institutional clustering was acknowledged as a limitation.

An undirected network was estimated via a Gaussian graphical model (GGM). The network structure was estimated using the extended Bayesian Information Criterion graphical least absolute shrinkage and selection operator (EBIC‐glasso) [34]. The estimation was based on a correlation matrix via the *“cor_auto”* function from the *“qgraph”* package, which automatically selects appropriate correlation measures based on variable distributions. The gamma hyperparameter was set to 0.5, following the recommendation of Foygel and Drton [35]. In this network, nodes represent variables, and edges represent regularized partial correlations, with thicker edges indicating stronger relationships. Node predictability, representing the proportion of variance in a node explained by its neighboring nodes (i.e., *R* ^2^), was visualized as a red shaded ring around each node, with a fuller ring indicating higher predictability [36]. Additionally, two key centrality indices were calculated to assess the importance of nodes, including expected influence (EI) and bridge expected influence (BEI). EI quantifies a node’s overall impact within the entire network, with higher EI values signifying greater importance. BEI evaluates a node’s cross‐community influence by assessing its cumulative effect on nodes belonging to different communities. In this study, BEI was employed to capture the connections between the emotional labor community and its three antecedent communities, reflecting the cross‐domain relationships proposed in our conceptual framework. Given that the network estimation was based on the correlation matrix, which is invariant to linear transformations, we used the original summed scores as input and did not perform standardization.

The accuracy and stability of the undirected network were assessed using the R package *“bootnet.”* First, 95% confidence intervals (CIs) for edge weights were estimated via nonparametric bootstrapping with 1000 resamples. Subsequently, the stability of EI and BEI was evaluated using case‐dropping bootstrap (1000 iterations). This procedure yielded correlation stability (CS) coefficients, which indicate the maximum proportion of cases that can be excluded while maintaining the stability of centrality indices. Following the recommendations of Epskamp et al., the values of CS coefficients should exceed 0.25 and preferably 0.50 to ensure a stable interpretation of the network [31].

A DAG was estimated using Bayesian network analysis, an approach that provides information regarding both the strength and the direction of probabilistic associations from cross‐sectional data. The network structure was learned using the hill‐climbing algorithm via the R package *“bnlearn”* [37], with the BIC‐g score. All variables were treated as Gaussian continuous variables. To evaluate structural robustness, we performed bootstrapping with 10,000 resamples (with replacement), estimating a network for each sample. The optimal inclusion threshold was determined automatically using the procedure implemented in the averaged.network() function of the bnlearn package, following Scutari and Nagarajan. The resulting threshold was 0.4593, and arcs with bootstrap strength values exceeding this threshold were retained in the averaged network [38]. The orientation of each retained edge was determined by its direction probability, defined as the proportion of bootstrap samples in which a specific direction appeared. The resulting direction probability for an edge reflects a probabilistic orientation tendency rather than a deterministic causal claim. In the final DAG, a directed edge from node A to node B was depicted if this orientation was present in more than 50% of the bootstrapped networks. Based on the rule of thumb, direction probabilities between 0.50 and 0.61 indicate weak directional support, while probabilities exceeding 0.70 suggest higher stability. As no prior constraints, blacklists, or whitelists were imposed during structure learning, the resulting network represents a purely data‐driven exploration of the associations among variables.

#### 2.4.3. Sensitivity Analysis

Given the potential influence of demographic and occupational factors on nurses’ emotional labor [39–41], we conducted two sensitivity analyses. Two undirected networks were reconstructed: one adjusting for demographic variables (age, gender, marital status, education level, and income status) and the other adjusting for occupational variables (work experience, hospital level, shift work, and department). Specifically, for each of the 11 nodes, a linear regression model was constructed with the node as the dependent variable and the corresponding covariates as independent variables. Standardized residuals were extracted and used to reconstruct the respective covariate‐adjusted networks [42]. The correlation coefficients between each covariate‐adjusted network and the original network were computed to assess the potential impact of demographic and occupational variables on the network structure.

## 3. Results

### 3.1. Sample Characteristics

Of the 2996 nurses included in the network, the average age was 34.79 years (SD = 8.87), and 2885 (96.3%) of them were female. Detailed demographic and occupational characteristics are shown in Table 1. Additionally, the descriptive statistics for the emotional labor, emotional display rule perceptions, perceived organizational support, and emotional intelligence are also summarized in Table 1.

**TABLE 1:** Demographic characteristics of participants (*n* = 2996).

Variables	Category	Range	*M* (*n*)	SD (%)
Age (years)			34.79	8.87
Gender	Male		111	3.7
	Female		2885	96.3

Marital status	Single		488	16.3
	Married		2409	80.4
	Other		99	3.3

Educational level	Junior college or below		240	8.0
	Undergraduate		2676	89.3
	Master or above		80	2.7

Income status	Insufficient		503	16.8
	Sufficient for essentials		1842	61.5
	More than sufficient		651	21.7

Work experience (years)			12.52	7.85
Hospital level	Tertiary		2873	95.9
	Secondary		123	4.1
Work shift	Daytime		1014	33.8
	Working in shifts		1982	66.2
Department	Medical ward		931	31.1
	Surgical ward		767	25.6
	Maternal and child health ward		391	13.1
	Emergency or intensive care ward		465	15.5
	Other		442	14.8

Emotional labor	Surface acting	7–35	22.49	5.58
	Deep acting	4–20	15.22	2.56
	Expression of naturally felt emotions	3–15	11.33	2.23
Emotional display rules	Positive display rules	4–20	17.52	2.26
	Negative display rules	3–15	12.63	2.12
Perceived organizational support	Emotional support	10–50	37.23	8.60
	Instrumental support	3–15	12.10	2.32
Emotional intelligence	Self‐emotion appraisal	0–24	18.58	3.63
	Others’ emotion appraisal	0–24	17.38	3.98
	Use of emotion	0–24	18.06	3.92
	Regulation of emotion	0–24	17.14	4.39

### 3.2. Undirected Network

**FIGURE 2:**
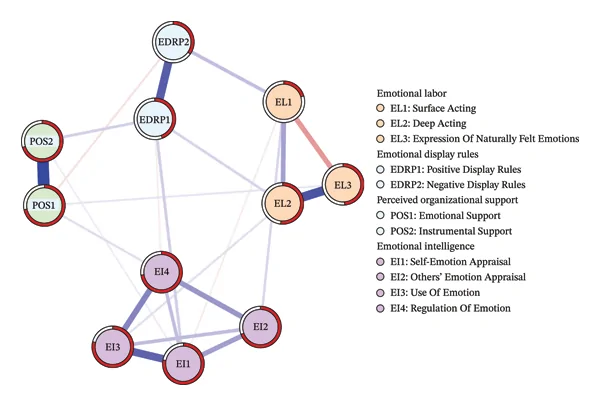
Undirected network of emotional labor, emotional display rules, perceived organizational support, and emotional intelligence among nurses. *Note*. Only edges with absolute weights greater than 0.05 are displayed to enhance visual clarity. Blue lines represent positive correlations, and red lines represent negative correlations. The red rings around the nodes indicate the predictability of this node.

**FIGURE 3:**
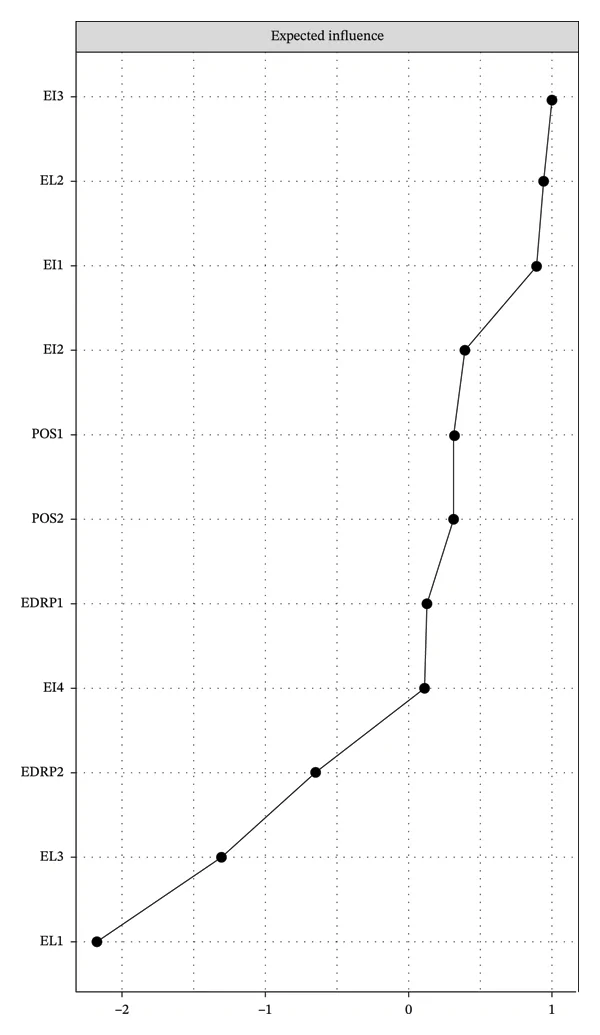
Expected influence of the undirected network. *Note.* EL1 = Surface Acting; EL2 = Deep Acting; EL3 = Expression of Naturally Felt Emotions; EDRP1 = Positive Display Rules; EDRP2 = Negative Display Rules; POS1 = Emotional Support; POS2 = Instrumental Support; EI1 = Self‐Emotion Appraisal; EI2 = Others’ Emotion Appraisal; EI3 = Use of Emotion; EI4 = Regulation of Emotion.

**FIGURE 4:**
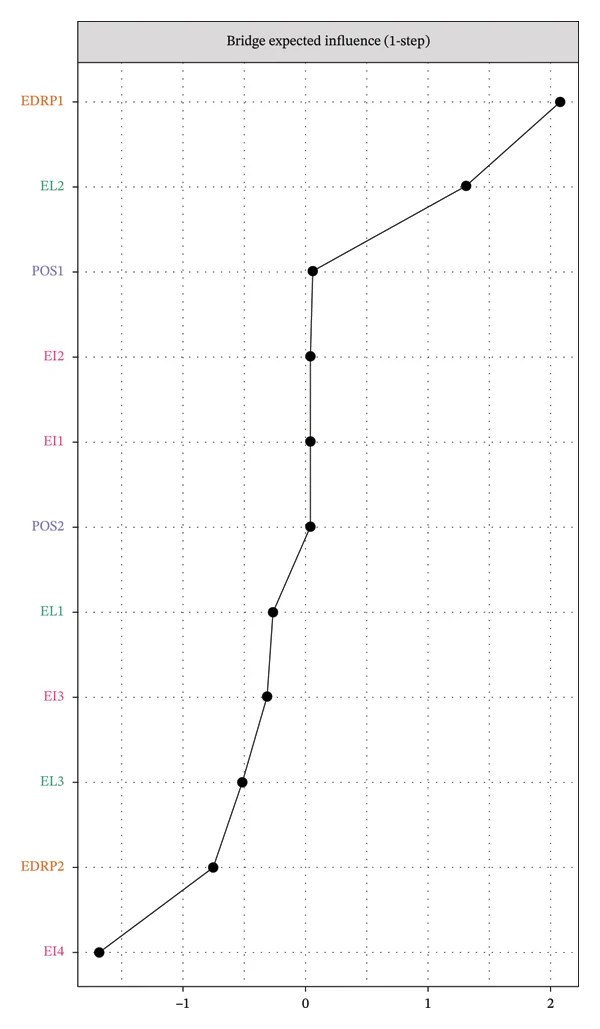
Bridge expected influence of the undirected network. *Note.* EL1 = Surface Acting; EL2 = Deep Acting; EL3 = Expression of Naturally Felt Emotions; EDRP1 = Positive Display Rules; EDRP2 = Negative Display Rules; POS1 = Emotional Support; POS2 = Instrumental Support; EI1 = Self‐emotion appraisal; EI2 = Others’ Emotion Appraisal; EI3 = Use of Emotion; EI4 = Regulation of Emotion.

The undirected network structure is depicted in Figure 2. The network density is represented by the proportion of nonzero edges. Out of 55 possible edges, 43 (78.2%) edges were evident in the final network, 33 of which were positive. For visual clarity, Figure 2 displays only edges with absolute edge weights greater than 0.05, while the complete network structure containing all 43 edges is presented in Figure S1. The strongest edges included the connection between “Emotional support” and “Instrumental support” (POS1–POS2, edge weight = 0.723). Detailed edge weights are provided in Table S1. In addition, node predictability values ranged from 0.204 to 0.789 across all nodes, with a mean of 0.582, indicating a relatively high degree of interconnectedness. Among all nodes, “Use of emotion” (EI3) showed the highest predictability (*R* ^2^ = 0.789), followed by “Self‐emotion appraisal” (EI1, *R* ^2^ = 0.779) and “Regulation of emotion” (EI4, *R* ^2^ = 0.727). The predictability estimates for all other nodes are provided in Table S2. The standardized estimates of EI and BEI are shown in Figures 3 and 4, respectively. “Use of emotion” (EI3) emerged as the most central node, followed by “Deep acting” (EL2) and “Self‐emotion appraisal” (EI1), with EI values of 1.131, 1.105, and 1.090, respectively. The BEI indices identified “Positive display rules” (EDRP1) as the most influential bridge node connecting different communities, with a BEI value of 0.360. The EI and BEI values for all nodes are provided in Table S3.

The stability estimation results for centrality indices are shown in Figure S2. The case‐dropping bootstrap procedure indicated that the EI and BEI values remained stable when dropping portions of the sample. The CS coefficients for both EI and BEI were 0.75, indicating excellent network stability. Figure S3 displays the bootstrapped 95% CIs for edge weights. The stronger edges (e.g., POS1–POS2) showed relatively narrower CIs, while the weaker edges (e.g., EL1–POS1) had wider CIs. Additionally, bootstrapped difference tests for centrality and edge weights are reported in Figures S4 and S5. The results indicated that the top‐ranked nodes (EI3, EL2, and EI1) differed statistically from others in EI, and EDRP1 showed significantly higher BEI than all other nodes. For edge weights, a considerable proportion of differences were statistically significant.

### 3.3. DAG

**FIGURE 5:**
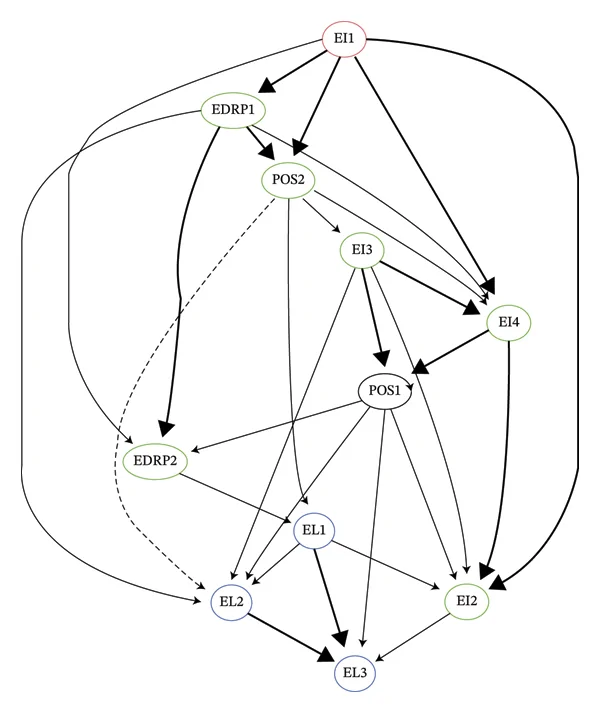
Directed network of emotional labor, emotional display rules, perceived organizational support, and emotional intelligence among nurses. *Note.* EL1 = Surface Acting; EL2 = Deep Acting; EL3 = Expression of Naturally Felt Emotions; EDRP1 = Positive Display Rules; EDRP2 = Negative Display Rules; POS1 = Emotional Support; POS2 = Instrumental Support; EI1 = Self‐Emotion Appraisal; EI2 = Others’ Emotion Appraisal; EI3 = Use of Emotion; EI4 = Regulation of Emotion. Red circle indicates the upstream node identified in the DAG; green circles indicate nodes directly connected to EI1 by directed edges; blue circles indicate nodes positioned downstream.

Figure 5 depicts the DAG derived from the Bayesian network analysis. The arrow thickness represents the change in the BIC upon removal of the arrow from the network. A larger absolute BIC value corresponds to a thicker arrow in Figure 5, indicating a greater contribution to the model structure. Within the DAG, “Self‐emotion appraisal” (EI1) showed a relatively upstream structural position, with potential directional associations to “Positive display rules” (EDRP1), “Negative display rules” (EDRP2), “Instrumental support” (POS2), “Use of emotion” (EI3), “Regulation of emotion” (EI4), and “Others’ emotion appraisal” (EI2). Additionally, “Surface acting” (EL1), “Deep acting” (EL2), and “Expression of naturally felt emotions” (EL3) were located relatively downstream within the estimated graph. Table 2 lists the BIC change values and direction probabilities for each arrow in Figure 5. The direction probabilities for most edges ranged from 0.50 to 0.63, indicating modest support for edge orientation. It should be noted that these probabilities reflect the relative stability of edge orientations across 10,000 bootstrapped samples, representing probabilistic tendencies within the cross‐sectional data rather than as evidence of temporal or causal ordering.

**TABLE 2:** Arrow weight in the directed acyclic graph.

Arrow		Value‐determining arrow thickness	
From	To	BIC	Direction probability
EL1	EL2	−27.99	0.59
EL1	EL3	−175.18	0.72
EL1	EI2	−32.79	0.81
EL2	EL3	−622.38	0.63
EDRP1	EL2	−38.94	0.61
EDRP1	EDRP2	−474.72	0.61
EDRP1	POS2	−56.20	0.50
EDRP1	EI4	−12.82	0.61
EDRP2	EL1	−97.54	0.61
POS1	EL2	−13.03	0.69
POS1	EL3	−13.47	0.71
POS1	EDRP2	−43.92	0.57
POS1	EI2	−2.84	0.66
POS2	EL1	−11.45	0.61
POS2	EL2	0.86	0.62
POS2	EI3	−32.60	0.51
POS2	EI4	−6.32	0.53
EI1	EDRP1	−294.47	0.60
EI1	EDRP2	−3.92	0.72
EI1	POS2	−268.52	0.57
EI1	EI2	−120.93	0.88
EI1	EI3	−1509.51	0.55
EI1	EI4	−131.89	0.66
EI2	EL3	−15.52	0.70
EI3	EL2	−90.41	0.73
EI3	POS1	−54.58	0.50
EI3	EI2	−46.61	0.78
EI3	EI4	−322.85	0.68
EI4	POS1	−56.64	0.52
EI4	EI2	−136.82	0.79

*Note:* EL1 = Surface Acting; EL2 = Deep Acting; EL3 = Expression of Naturally Felt Emotions; EDRP1 = Positive Display Rules; EDRP2 = Negative Display Rules; POS1 = Emotional Support; POS2 = Instrumental Support; EI1 = Self‐Emotion Appraisal; EI2 = Others’ Emotion Appraisal; EI3 = Use of Emotion; EI4 = Regulation of Emotion.

### 3.4. Sensitivity Analysis

Figures S6 and S7 present the complete network structures after adjusting for demographic and occupational variables, respectively. The Pearson correlations between the edge weight matrices of the original network and the covariate‐adjusted networks were high (for demographic adjustment: *r* = 0.9998, *p* < 0.001; for occupational adjustment: *r* = 0.9999, *p* < 0.001), indicating that the network structure remained stable after adjusting for these covariates.

## 4. Discussion

This study employed two network analyses to explore the complex relationships among emotional labor and its three antecedents among nurses. Results from the undirected network highlighted “Use of emotion” and “Deep acting” as the most central nodes, and “Positive display rules” as a critical bridge node. Additionally, findings from both the undirected network and the DAG revealed the prominent role of “Self‐emotion appraisal” within nurses’ emotional labor network.

Specifically, in the undirected network, “Use of emotion” exhibited the highest EI, indicating that it was strongly connected with other components and occupied a structurally central position within the estimated emotional labor network. UOE refers to the capability to channel emotions toward constructive performance, enabling individuals to transform emotional resources into work motivation and goal‐oriented behaviors [28, 43]. According to the conservation of resources theory, individuals can enrich their resources by investing those they already possess or can access in the environment [44]. In this study, the high centrality of “Use of emotion” may be explained as follows: Nurses who score highly on this dimension may have a more integrated process for handling emotions and may more effectively leverage organizational support, thereby facilitating appropriate emotional expressions to patients [45]. Additionally, research on academic settings has shown that, among the four dimensions of emotional intelligence, only UOE exerts a broadly positive influence on performance outcomes, which is similar to our finding. Therefore, we propose that the ability to use emotion may be a key competency in nurses’ management of emotional labor [46].

“Deep acting” also exhibited high EI, indicating its strong connectivity with other nodes within the entire network. This aligns with previous studies demonstrating that emotional display rules, emotional intelligence, and perceived organizational support are closely associated with deep acting [10, 13, 47]. Unlike Surface Acting and the Expression of Naturally Felt Emotions, Deep Acting involves aligning one’s inner feelings with organizational expectations through attentional deployment or cognitive reappraisal. This process requires the mobilization of available personal and organizational resources [48–50]. Notably, such mobilization does not necessarily lead to excessive cognitive depletion but can represent a synergistic integration of internal and external factors [51]. From a theoretical perspective, the psychological congruence associated with deep acting may contribute to positive affective experiences, which previous studies have linked to lower emotional exhaustion and further resource gain [52, 53].

“Positive display rules” exhibited the highest BEI, indicating a structurally important bridging position across the domains of display rules, emotional intelligence, perceived organizational support, and emotional labor. This bridging position may reflect the close associations of positive display rules with both psychological and organizational resources involved in emotional labor. Previous studies have suggested that positive display rules may be associated with more positive emotional experiences and supportive responses from colleagues and supervisors [54, 55]. Such interactions have also been associated with lower emotional exhaustion and higher job satisfaction [56].

Nevertheless, these centrality and bridge centrality findings should be interpreted as indicators of structural connectivity rather than causal importance or intervention priority. They may also be influenced by conceptual breadth, measurement overlap, shared method variance, and differences in scale properties.

The DAG structure suggested that “Self‐emotion appraisal” occupied the most upstream structural position, with the three emotional labor strategies located relatively downstream and “Expression of naturally felt emotions” appearing at the terminal position within the estimated graph. This structure was broadly consistent with our hypothesized framework based on Grandey’s emotional regulation model, in which antecedent‐related factors are theoretically positioned before emotional labor strategies. However, given the cross‐sectional design and the modest direction probabilities for several edges, this pattern should be interpreted as a probabilistic structural tendency rather than evidence of temporal or causal ordering. The terminal position of “Expression of naturally felt emotions” may be theoretically understandable because this strategy represents a state of spontaneous congruence that requires no further deliberate regulatory effort [6]. This interpretation is consistent with the perspective of Chen et al., who proposed that the expression of naturally felt emotions may represent a later stage of the emotional labor process [57].

Notably, both network analyses highlighted the structural relevance of “Self‐emotion appraisal.” Its high EI in the undirected network indicated that it was strongly connected with several other nodes, while in the estimated DAG it occupied a relatively upstream structural position. This pattern is broadly consistent with the cascading model of emotional intelligence, which proposes that emotion perception and understanding precede more complex emotion‐related processes [58]. Supporting this theoretical ordering, Pekaar et al. reported in a weekly diary study that SEA predicted subsequent self‐emotion regulation behaviors [59]. Taken together, the present findings suggest that SEA may occupy an early and structurally relevant position within the emotional labor network. However, because the current data are cross‐sectional and several edge directions received only modest support, this interpretation remains exploratory. The identified orientation pattern therefore provides testable hypotheses regarding possible temporal relationships for future longitudinal research rather than evidence of established causal pathways.

### 4.1. Implications for Nursing Management

These findings suggest that the interplay among emotional labor, display rules, emotional intelligence, and perceived organizational support may be better understood from a comprehensive and synergistic perspective. Because the present findings were derived from an exploratory cross‐sectional network analysis, the following recommendations should be interpreted as potential management considerations rather than established intervention effects. Nursing managers may consider paying greater attention to nurses’ emotional labor by creating conditions that support adaptive emotional regulation and allow naturally felt emotions to be expressed appropriately. These management considerations are derived from a predominantly female sample of clinical nurses working mainly in tertiary hospitals in Shandong Province, China, and should therefore be applied cautiously beyond similar healthcare contexts.

First, UOE and SEA may represent relevant competencies for future emotional intelligence–related management programs because of their structurally important positions in the network. In practice, managers may consider simulation‐based communication training to help nurses recognize their own emotions and translate them into effective communication behaviors [60]. Regular structured reflective debriefing may also support nurses in examining challenging emotional responses and developing more adaptive emotional regulation strategies in clinical practice [61]. However, the effectiveness of these approaches should be evaluated in longitudinal or intervention studies.

Second, the bridging position of positive display rules suggests that constructive emotional norms may be relevant to ward climate and individual regulation. Managers may consider establishing clear, flexible, and supportive expectations for professional emotional expression. Importantly, supportive positive norms should be distinguished from coercive emotional display requirements. Rigid expectations that require nurses to continuously display positivity or suppress legitimate distress may increase surface acting, emotional suppression, and emotional dissonance. Therefore, clinical units should also provide psychologically safe channels through which nurses can express negative emotions appropriately and seek support.

Third, a supportive work environment remains an important consideration in nursing management. Managers may strengthen instrumental support, such as staffing adequacy, protected breaks, and supervisor availability, as well as emotional support through peer support, active listening, and empathic communication [62, 63].

Finally, feasible indicators may be incorporated into routine quality and workforce monitoring. These may include burnout warning signs, periodic assessment of emotional exhaustion, and turnover intention, which may support the earlier identification of staff who could benefit from additional assistance. The usefulness of these management approaches should be further examined through prospective and intervention‐based research.

### 4.2. Limitations

Several limitations in this study should be acknowledged. First, all variables were assessed through self‐report questionnaires and incorporated into the network as summed scores. This approach may introduce measurement error and inflate associations due to shared method variance, which can bias the estimated edge weights. Second, participants were recruited from a single province in China, and the sample was predominantly female and drawn from tertiary hospitals. Although our preliminary analysis indicated that clustering within hospital levels and departments was negligible, the potential impact of unmodeled nesting at other levels (e.g., cities) cannot be entirely ruled out. Thus, the results may be most applicable to similar hospital contexts and staffing models. Future replication in gender‐balanced samples and across different provinces and settings is needed. Third, the application of EBIC‐glasso regularization inherently favors sparsity. Although this approach reduces false positives, it may result in the omission of weaker yet theoretically relevant edges. Fourth, while our sensitivity analysis showed that the network structure was robust to adjustment for demographic and occupational factors, other unmeasured variables (e.g., job title, workload) may still influence multiple nodes and thereby contribute to spurious associations. Finally, due to the cross‐sectional design, the DAG results represent probabilistic associations rather than temporal causality. The marginal bootstrap support for several edge orientations suggests that these directions should be interpreted as preliminary tendencies rather than established pathways. In addition, the exact number of participating hospitals and hospital‐ and city‐specific identifiers were not retained in the anonymized dataset. Therefore, clustering at the individual hospital and city levels could not be directly examined, and the response rate could not be calculated because the total recruitment denominator was unavailable. These factors may limit the assessment of selection bias and institutional‐level variability. Hospital‐ and city‐specific identifiers were not retained in the anonymized dataset; therefore, clustering within individual hospitals, cities, wards, or clinical units could not be directly evaluated. Although variation across hospital‐level and department categories was minimal, residual institutional clustering cannot be ruled out and may have affected the estimated network structure.

In summary, these limitations indicate that the present findings should be viewed as preliminary and exploratory. Future research should employ longitudinal design across diverse cultural and organizational contexts or utilize experience sampling methods to validate the robustness and generalizability of the network structure identified in this study.

## 5. Conclusion

This study provides preliminary insights into the interrelationships among emotional labor, emotional display rules, emotional intelligence, and perceived organizational support among clinical nurses in Shandong Province, China, from a network perspective. The findings suggest that these constructs were organized as an interconnected network rather than functioning as isolated variables. In the estimated DAG, “Self‐emotion appraisal” occupied a relatively upstream structural position, whereas “Use of emotion” and “Deep acting” showed high EI and occupied structurally central positions in the undirected network. “Positive display rules” exhibited the highest bridge‐expected influence, indicating a structurally important connection between organizational norms and individual emotion regulation. These findings may inform the future development and evaluation of emotional intelligence–related programs, supportive emotional norms, and organizational support strategies. However, given the cross‐sectional and exploratory design, the identified nodes should not be interpreted as confirmed causal factors or intervention targets. As the sample was predominantly female and drawn mainly from tertiary hospitals in one Chinese province, the findings are most directly applicable to clinical nurses in similar Chinese tertiary hospital contexts and require replication in more diverse regions, hospital levels, and cultural settings.

## Author Contributions

Xu‐Dong He analyzed and interpreted data and wrote the draft. Xiao‐He Lin wrote the draft and participated in writing–reviewing and editing. Xiang‐Yu Zhao, Xiao‐Na Shen, and Guo‐Peng Li participated in investigation, data curation. Ping Li developed idea and concepts for the study and participated in writing–reviewing and editing.

Xu‐Dong He and Xiao‐He Lin contributed equally.

## Funding

This research received no specific grant from any funding agency in the public, commercial, or not‐for‐profit sectors.

## Ethics Statement

The original study was approved by the institutional review board of the School of Nursing and Rehabilitation, Shandong University, Jinan, Shandong Province, China (2020‐R‐135), and the approval covered subsequent analyses of anonymized data. Participants were informed that their de‐identified data could be used in future related research, and completion and submission of the online questionnaire indicated informed consent. The present secondary analysis involved only anonymized data and no additional contact with participants.

## Conflicts of Interest

The authors declare no conflicts of interest.

## Supporting Information

Additional supporting information can be found online in the Supporting Information section.

## Supporting information


**Supporting Information** Details on the edge weights of the undirected network, along with stability, accuracy, difference tests, and sensitivity analyses are included in the Supporting File.

## Data Availability

The data that support the findings of this study are available on request from the corresponding author. The data are not publicly available due to privacy or ethical restrictions.
