# Supplementary material for: Network Analysis of Emotional Labor, Emotional Display Rules, Perceived Organizational Support, and Emotional Intelligence Among Nurses
Source: J Nurs Manag. 2026 Aug 19;2026:4202514. doi: 10.1155/jonm/4202514 (PMC13488373; doi:10.1155/jonm/4202514)
Supplement: Supplementary file 1 [file JONM-2026-4202514-s001.docx]

**Table S**[**1**](https://link.springer.com/article/10.1186/s12888-023-04971-x#MOESM1). Weighted adjacency matrix.

|  | EL1 | EL2 | EL3 | EDRP1 | EDRP2 | POS1 | POS2 | EI1 | EI2 | EI3 | EI4 |
| --- | --- | --- | --- | --- | --- | --- | --- | --- | --- | --- | --- |
| EL1 | - |  |  |  |  |  |  |  |  |  |  |
| EL2 | 0.261 | - |  |  |  |  |  |  |  |  |  |
| EL3 | -0.291 | 0.550 | - |  |  |  |  |  |  |  |  |
| EDRP1 | 0 | 0.120 | 0.006 | - |  |  |  |  |  |  |  |
| EDRP2 | 0.182 | 0 | -0.021 | 0.488 | - |  |  |  |  |  |  |
| POS1 | -0.008 | 0.042 | 0.071 | 0 | -0.080 | - |  |  |  |  |  |
| POS2 | -0.045 | 0.038 | 0 | 0.133 | 0 | 0.723 | - |  |  |  |  |
| EI1 | -0.057 | 0.010 | 0.0001 | 0.135 | 0.044 | 0 | 0.052 | - |  |  |  |
| EI2 | 0.113 | 0 | 0.029 | 0.007 | 0 | 0.036 | 0 | 0.270 | - |  |  |
| EI3 | 0 | 0.083 | 0.015 | 0.010 | 0 | 0.031 | 0.014 | 0.456 | 0.185 | - |  |
| EI4 | -0.026 | 0 | 0.036 | -0.052 | -0.009 | 0.094 | -0.008 | 0.181 | 0.293 | 0.336 | - |

*Note.* EL1 = Surface acting; EL2 = Deep acting; EL3 = Expression of naturally felt emotions; EDRP1 = Positive display rules; EDRP2 = Negative display rules; POS1 = Emotional support; POS2 = Instrumental support; EI1 = Self-emotion appraisal; EI2 = Others’ emotion appraisal; EI3 = Use of emotion; EI4 = Regulation of emotion.

**Table S2**. Node predictability (R²) for all nodes.

|  | R^2^ |
| --- | --- |
| EL1 | 0.204 |
| EL2 | 0.516 |
| EL3 | 0.487 |
| EDRP1 | 0.442 |
| EDRP2 | 0.347 |
| POS1 | 0.717 |
| POS2 | 0.702 |
| EI1 | 0.779 |
| EI2 | 0.693 |
| EI3 | 0.789 |
| EI4 | 0.727 |

*Note.* EL1 = Surface acting; EL2 = Deep acting; EL3 = Expression of naturally felt emotions; EDRP1 = Positive display rules; EDRP2 = Negative display rules; POS1 = Emotional support; POS2 = Instrumental support; EI1 = Self-emotion appraisal; EI2 = Others’ emotion appraisal; EI3 = Use of emotion; EI4 = Regulation of emotion.

**Table S3**. Expected influence and bridge expected influence centrality estimates for all nodes.

|  | EI | BEI |
| --- | --- | --- |
| EL1 | 0.129 | 0.158 |
| EL2 | 1.105 | 0.293 |
| EL3 | 0.395 | 0.136 |
| EDRP1 | 0.848 | 0.360 |
| EDRP2 | 0.603 | 0.115 |
| POS1 | 0.909 | 0.185 |
| POS2 | 0.907 | 0.184 |
| EI1 | 1.090 | 0.184 |
| EI2 | 0.932 | 0.184 |
| EI3 | 1.131 | 0.154 |
| EI4 | 0.845 | 0.036 |

*Note.* EI = Expected influence; BEI = Bridge expected influence; EL1 = Surface acting; EL2 = Deep acting; EL3 = Expression of naturally felt emotions; EDRP1 = Positive display rules; EDRP2 = Negative display rules; POS1 = Emotional support; POS2 = Instrumental support; EI1 = Self-emotion appraisal; EI2 = Others’ emotion appraisal; EI3 = Use of emotion; EI4 = Regulation of emotion.

**Figure S1.**Undirected network of emotional labor, emotional display rules, perceived organizational support and emotional intelligence among nurses.

*Note.* This figure displays all retained edges (43 out of 55 possible). Blue lines represent positive correlations, and red lines represent negative correlations. The red rings around the nodes indicate the predictability of this node.

**Figure S2.** Stability of centrality indices as assessed using the case-dropping bootstrap method.

*Note.* The x-axis represents the percentage of cases of the original sample used at each step. The y-axis represents the average of correlations between the centrality indices from the original network and the centrality indices from the networks that were re-estimated after excluding increasing percentages of cases.

**Figure S3.** Accuracy of edge weights for the current network model.

*Note.* The gray shaded areas represent the 95% Confidence Intervals of edge weights, estimated using the non-parametric bootstrap procedure. Narrower gray areas indicate higher estimation precision, whereas wider areas indicate lower precision. The black line denotes the mean edge weight from the bootstrap samples, and the red line denotes the original sample values. Edges are ordered from the highest to the lowest mean edge weight.

EL1 = Surface acting; EL2 = Deep acting; EL3 = Expression of naturally felt emotions; EDRP1 = Positive display rules; EDRP2 = Negative display rules; POS1 = Emotional support; POS2 = Instrumental support; EI1 = Self-emotion appraisal; EI2 = Others’ emotion appraisal; EI3 = Use of emotion; EI4 = Regulation of emotion.

**Figure S4.** Estimation of node centrality difference by bootstrapped difference test.

*Note.* Figure S3A presents results for expected influence (EI) and Figure S3B presents results for bridge expected influence (BEI). Gray boxes indicate nodes that do not significantly differ from one another, while black boxes represent nodes that do differ significantly (α = 0.05). White boxes show the estimated centrality values.

EL1 = Surface acting; EL2 = Deep acting; EL3 = Expression of naturally felt emotions; EDRP1 = Positive display rules; EDRP2 = Negative display rules; POS1 = Emotional support; POS2 = Instrumental support; EI1 = Self-emotion appraisal; EI2 = Others’ emotion appraisal; EI3 = Use of emotion; EI4 = Regulation of emotion.

**Figure S5.** Estimation of edge weights difference by bootstrapped difference test.

*Note.* Gray boxes indicate edges that do not significantly differ from one another, while black boxes represent edges with significant difference from one another (α = 0.05). Blue and orange boxes on the diagonal correspond to edge-weights with positive and negative correlations, respectively.

EL1 = Surface acting; EL2 = Deep acting; EL3 = Expression of naturally felt emotions; EDRP1 = Positive display rules; EDRP2 = Negative display rules; POS1 = Emotional support; POS2 = Instrumental support; EI1 = Self-emotion appraisal; EI2 = Others’ emotion appraisal; EI3 = Use of emotion; EI4 = Regulation of emotion.

**Figure S6.** Undirected network of emotional labor, emotional display rules, perceived organizational support and emotional intelligence adjusted for demographic variables.

*Note.* Blue lines represent positive correlations, and red lines represent negative correlations. The red rings around the nodes indicate the predictability of this node. The network was adjusted for age, gender, marital status, education level and income status.

**Figure S7.** Undirected network of emotional labor, emotional display rules, perceived organizational support and emotional intelligence adjusted for occupational variables.

*Note.* Blue lines represent positive correlations, and red lines represent negative correlations. The red rings around the nodes indicate the predictability of this node. The network was adjusted for work experience, hospital level, shift work, and department.
